# Relationships Among Soda and Energy Drink Consumption, Substance Use, Mental Health and Risk-Taking Behavior in Adolescents

**DOI:** 10.3390/children11121448

**Published:** 2024-11-27

**Authors:** Surya Suresh, Jennifer L. Temple

**Affiliations:** 1 Department of Exercise and Nutrition Sciences, University at Buffalo, Buffalo, NY 14214, USA; suryasur@buffalo.edu; 2 Department of Community Health and Health Behavior, University at Buffalo, Buffalo, NY 14214, USA

**Keywords:** caffeine, energy drinks, substance use, risk taking, adolescent, mental health

## Abstract

**Background/Objectives**: Energy drink (ED) use is increasing among children and adolescents, but little is known about the impacts on health, including substance use and mental health. The purpose of this study was to examine the relationship between soda and ED consumption and substance use, mental health, and risk taking in a nationally representative sample of high school students. **Methods:** We used data from the 2019 Youth Risk Behavior Surveillance System (YRBS) from New Jersey, Montana, and Florida to assess these relationships using binary and multinomial regression analyses to determine odds ratios, comparing non-consumers with daily consumers. The sample was 10,548 adolescents (51.6% female) between the ages of 13–19 years. **Results**: Daily soda and ED consumption were associated with greater odds of substance use (OR(95% CI): 5.8 (3.7, 6.9)/10.2 (6.4, 16.3)), poorer mental health (OR(95% CI): 2.6 (1.3, 4.8)/1.8 (1.2, 2.8), and higher odds of eating fast food (OR(95% CI): 17.2 (8.9, 33)/10.6 (5.6, 19.9). These effects were moderated by sex. **Conclusions**: These findings suggest that soda and ED use are associated with greater risk taking among adolescents and that these relationships are moderated by sex. Future studies should determine the directionality of these relationships and examine the impact of reduced soda and ED consumption on health behaviors in children and adolescents.

## 1. Introduction

Caffeine use is prevalent in the US diet, with 87% of adults consuming it daily, averaging 193 mg (1.2 mg/kg body weight) [1]. In children, caffeine intake is far lower, with the average being around 50 mg/day in adolescents [2], and the primary source is carbonated soda [2]. Although energy drinks (ED) contributed to a relatively smaller proportion of the total caffeine intake, ED consumption is growing, in particular among adolescents [3]. While there are some benefits of caffeine in terms of attention and mental alertness [4], overconsumption can lead to insomnia, anxiety, and physical discomfort [5]. Caffeine intake can be especially concerning in children and adolescents, and concerns are growing due to the popularity of highly caffeinated beverages, such as EDs [6,7]. EDs are considered dietary supplements and, therefore, have no limit on the amount of caffeine that can be added. The average ED contains about 200 mg of caffeine, but the amount varies widely from as little as 85 mg to over 400 mg. The US Food and Drug Administration recognizes caffeine as safe for adults in amounts < 400 mg/day, but recommendations differ for children, with 100 mg limits for children 12 and older and no caffeine consumption for children under the age of 12 years [6].

Children and adolescents may be particularly vulnerable to the harmful effects of caffeine, especially in the form of EDs. The review by Soós et al. concluded that there are no safe dosages described regarding caffeine or ED consumption for children [8]. Similarly, Pollak et al. found that caffeine intake among 7th to 9th graders ranged between 0 and 800 mg/d, with an average of 62.7 mg/d, and that higher caffeine intake was associated with shorter nocturnal sleep duration, increased wake time after sleep onset, and increased daytime sleep [9]. Another study by Richards and Smith (2016) found that high caffeine consumption (i.e., 1000 mg/week) was associated with low general health in secondary school children [10]. Although adolescent caffeine intake does not appear to be increasing over time [11], the proportion of caffeine intake represented by coffee and EDs has increased and that represented by soda intake has declined [12]. Similarly, Vercammen et al. (2019) found that from 2003 to 2016, the prevalence of ED consumption increased significantly for adolescents, young adults, and middle-aged adults [13]. Lastly, the study by Tran et al. found that, based on NHANES data from 2003 to 2012, almost 85% of US teenagers (ages 13–17), young adults (ages 18–24), and adults (ages 25–29) reported consuming caffeine [14].

Daily caffeine intake is significantly higher for males, older people, smokers, and those showing higher scores on impulsivity, sensation seeking, and a facet of reward sensitivity [15,16]. This suggests that caffeine intake may be related to decision-making processes that lead to higher risk-taking behaviors among adolescents. Previous studies in college students showed a strong relationship between ED consumption and risk-taking behavior, in particular in males [17,18], but these relationships have not been examined in younger adolescents. A recent meta-analysis found that ED consumption was associated with greater violent and risky behaviors, “junk food” intake, and polysubstance use, including tobacco and alcohol [19]. In addition, a previous study from our laboratory showed that daily soda consumption was associated with greater risk taking among adolescents when compared with non-soda consumers in the 2011 YRBS [20]. Our prior analysis was limited to soda, as the survey did not include EDs at that time. Finally, previous studies have not examined relationships between soda and ED consumption and newly emerging risk behaviors, such as vaping, and few have examined mental health outcomes in the same study populations.

The objective of this research was to extend the findings of our previous study [19] by investigating the associations between soda and ED consumption and risk-taking behavior, including behaviors not previously studied, such as vaping and mental health outcomes. We hypothesized that the daily consumption of sodas and EDs would be associated with greater risk-taking behavior in high school students compared to no consumption. To examine this hypothesis, we analyzed data from the 2019 YRBS, comparing the likelihood of engaging in various risk-taking behaviors among students who reported daily or no soda and ED intake.

## 2. Materials and Methods

### 2.1. Study Sample

For these analyses, we used data from the 2019 Youth Risk Behavior Surveillance System (YRBS), which is an annual survey of risk-taking and other behaviors in US high school students sponsored biennially by the Centers for Disease Control and Prevention (CDC; [21]). While there is a standard set of questions asked on all surveys, each state is allowed to individualize the survey. Questions about ED use were only asked in three states, Montana, New Jersey, and Florida. We, therefore, limited our analyses to participants from these states for a total of 10,548 teens. The CDC Institutional Review Board approved the protocol for national data collection [21]. The methods of the YRBS data collection have been published previously and have been shown to be reliable and valid [22,23].

The primary goal of our analysis was to determine differences in the odds of engaging in certain risk-taking and health behaviors as a function of beverage consumption. Our two predictor variables in this analysis were soda and ED consumption. Adolescents were asked the following questions: “During the past 7 days, how many times did you drink a can, bottle, or glass of soda or pop, such as Coke, Pepsi, or Sprite?” and “During the past 7 days, how many times did you drink a can, bottle or glass of ED, such as Red Bull or Jolt?”. The choices for both of these questions were “I did not drink ___ in the past 7 days, 1–3 times, 4–6 times, 1 time per day, 2 times per day, 3 times per day, 4 or more times per day”. We used these answers to create three categories for soda and ED consumption: No consumption, occasional consumption (1–6 times in a week), and daily consumption (1 or more times per day). For the sake of consistency, all comparisons shown are between “No” intake and “Daily” intake. Our dependent measures in these analyses included illicit substance use behavior, such as cigarette smoking, vaping, alcohol use, marijuana use, and prescription drug use; violent behaviors, including bringing a weapon to school and engaging in physical fights; mental health outcomes, including feeling sad or hopeless, reporting mental health as “not good”, and suicide attempts; and general lifestyle behaviors, including sleep, physical activity, fast food, and fruit and vegetable intake. Some of the questions related to mental health were not asked in every state, so the number of teens responding was lower.

### 2.2. Analytic Plan

Study sample demographics were analyzed using Chi-squared and ANOVA with soda consumption as the between subjects variable. We included sex, age, race/ethnicity, grade, BMI percentile, estimated daily caffeine consumption (from soda and EDs), and hours of sleep on weeknights. Sex was assessed with the question “what is your sex?” and the options were female, male, or missing. We chose to use the term “sex” throughout the paper, as that was the term used in the question. We used binary and multinomial regression analyses to determine if daily soda and ED consumption predicted our various risk-taking behavior. We used sex and age as a covariate in all analyses, since consumption of soda and ED increases as a function of age and differs as a function of sex. Response options differed across the dependent measures, so they were recoded to fit into 5 levels of analysis (1 being never and 5 being the maximum number of times in the time frame offered). Each model was run twice, once without sex as a predictor and once with sex as a predictor to determine the impact of sex. All data were analyzed using SPSS 27.0 and data were considered significant if *p* < 0.05. All data shown are the fully adjusted models. Missing data were not included in the analyses.

## 3. Results

### 3.1. Sample Characteristics

The total sample size for this analysis was 10,548, with 51.6% female and 48.4% male. Respondents were fairly evenly divided among grade level, with the majority being aged 15–17 years. About 50% of the sample was non-Hispanic white with a BMI percentile of 61.6 kg/m^2^, an average daily caffeine intake of 64.5 mg, and average hours of sleep on school nights of 6.5 h. Table 1 shows the characteristics for the entire study population as well as the data divided by soda consumption condition. There were significant differences as a function of soda consumption frequency for all variables except BMI percentile (Table 1).

### 3.2. Relationship Among Soda Consumption and Substance Use Behaviors

We found significantly higher odds of engaging in substance use behaviors in daily vs. no soda consumers (Table 2). Thirty percent of the sample reported vaping at least once in the past 30 days. Individuals who consumed soda daily were 7.3 times more likely (CI 4.8, 11.1; *p* < 0.0001) to have reported vaping every day and 1.9 times more likely (CI 1.3, 2.8; *p* < 0.0001) to have reported vaping at least one time as compared to soda non-consumers. Similarly, individuals who consumed soda daily were 6.4 times more likely (CI 3.7, 11.0; *p* < 0.0001) to have reported drinking alcohol every day and 1.3 times more likely (CI 1.1, 1.5; *p* < 0.0001) to have reported drinking alcohol at least one time in the past month as compared to soda non-consumers.

### 3.3. Relationship Among ED Consumption and Substance Use Behaviors

We conducted a similar analysis for ED consumption and found a similar pattern (Table 2), with daily ED consumption associated with higher odds of substance use when compared with no EDs. Individuals who consumed EDs daily were 10.9 times more likely (Cl 6.8, 17.5; *p* < 0.001) to have reported vaping every day and 2.8 times more likely (Cl 1.6, 4.9; *p* < 0.001) to have reported vaping at least once in the last 30 days when compared to ED non-consumers. In addition, individuals who consumed EDs daily were 19.5 times more likely (Cl 11.4, 33.6; *p* < 0.001) to have reported alcohol consumption every day for the past 30 months as compared to ED non-consumers.

### 3.4. Relationship Among Soda and ED Consumption and Violent Behaviors

For this analysis, we examined daily vs. none for both soda and ED consumption and assessed the relationship with self-reported “Brought a weapon to school” and “Been in a physical fight” in the past 30 days. We found that both daily soda and daily ED consumption increased the odds of bringing a weapon to school as well as being in a physical fight. Individuals who consumed soda daily were 3.1 times more likely (Cl 2.5, 3.9; *p* < 0.001) to have reported bringing a weapon to school, whereas individuals who consumed EDs daily were 2.1 times more likely (Cl 1.8, 2.5; *p* < 0.001) to have reported bringing a weapon to school in the past month. On the other hand, individuals who consumed soda every day were 5.1 times more likely (Cl 3.5, 7.6; *p* < 0.001) to have reported being in a physical fight at least 12 times and 2.6 times more likely (Cl 2.2, 3.1; *p* < 0.001) to have reported being in a physical fight at least once in the past 30 days. Individuals who consumed EDs daily were 2.6 times more likely (Cl 1.9, 3.6; *p* < 0.001) to have reported being in a physical fight at least 12 times and 2.2 times more likely (CI 1.9, 2.5; *p* < 0.001) to have reported being in a physical fight at least once in the past month as compared to ED non-consumers.

### 3.5. Relationship Among Soda and ED Consumption and Mental Health Outcomes

For this analysis, we examined daily vs. none for both soda and ED consumption and assessed the relationships with self-reported “Felt sad or hopeless almost every day for two weeks in the past 12 months”, number of suicide attempts in past 12 months, and “Mental Health Not Good” in past 30 days (Table 3). We found that both daily soda and daily ED consumption increased the odds of feeling sad or hopeless for 2 weeks in the past year and the number of suicide attempts reported. Individuals who consumed soda every day were 1.3 times more likely (Cl 1.1, 1.5; *p* < 0.001) to have been sad or hopeless every day for two weeks in the past 12 months, whereas there was no significant relationship between daily ED consumption and reports of feeling sad or hopeless every day for the past 12 months (*p* > 0.05). However, individuals who consumed soda everyday were 5.5 times more likely (Cl 2.8, 10.7; *p* < 0.001) to have attempted suicide at least six times in the past 12 months and individuals who consumed EDs were 3.1 times more likely (Cl 1.8, 5.3; *p* < 0.001) to have attempted suicide at least six times in the past 12 months. There were no relationships between reporting “Mental health not good in past 30 days” and soda or ED consumption (all *p* > 0.05).

### 3.6. Relationship Among Soda and ED Consumption and Lifestyle Behaviors

For this analysis, we examined daily vs. none for both soda and ED consumption and assessed the relationship with self-reported average hours of sleep on school nights, servings of fruit eaten per week, servings of vegetables eaten per week, fast food consumed during the week, and number of days each week they carried out at least an hour of physical activity (Table 4). We found that daily soda and daily ED consumption increased the odds of fast-food consumption at least 7 times during the week. There was no significant relationship between daily soda consumption and average hours of sleep on school nights, fruit consumption per week, vegetable consumption per week, and physical activity (*p* > 0.05 for all). However, people who consumed soda were 17.5 times more likely (Cl 11.2, 27.4; *p* < 0.001) and individuals who consumed EDs daily were 4.9 times more likely (Cl 3.45, 6.94; *p* < 0.001) to have consumed fast food at least 7 times in the past week. There was no significant relationship between ED consumption and nightly sleep duration or ED consumption and vegetable consumption per week. However, there was a relationship between ED consumption and fruit consumption per week and physical activity, with daily ED consumption associated with higher fruit consumption, but an increase of 2.7 times more likely (CI 2.3, 3.1; *p* < 0.001) to not engage in any physical activity compared to ED non-consumers.

### 3.7. Sex Differences in Soda and ED Consumption and the Relationships with Other Behaviors

**Figure 1:**
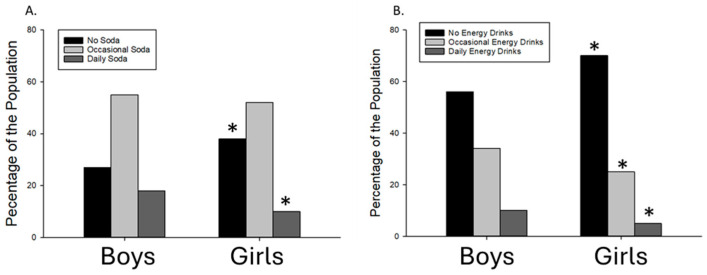
Soda (**A**) and energy drink (**B**) consumption in boys (left set of bars) and girls (right set of bars). A higher percentage of girls were soda and energy drink non-consumers compared to boys, and a lower percentage of girls were daily consumers compared to boys. For energy drinks, girls were also less likely to be occasional consumers compared to boys. All *p* < 0.05. * = significantly different from Boys.

For both soda consumption and ED consumption, girls were more likely to be non-consumers than boys and less likely to be daily consumers when compared to boys (Figure 1). For EDs, girls were also less likely to be occasional consumers than boys.

When we examined the relationships among sex, soda consumption, and engaging in different behaviors, we found sex differences in all of them. However, for several of the behaviors (alcohol use, being in a physical fight, and consuming fast food), if girls were daily soda consumers, they had greater odds of engaging in these behaviors compared to boys with the same level of soda use (Table 5). Girls who consumed soda everyday were 17.8 times more likely (Cl 5.0, 62.9; *p* < 0.001) to have consumed alcohol every day in the past month compared to boys, who were only 3.3 times more likely (Cl 1.8, 6.2; *p* < 0.001) to have consumed alcohol every day for the past month. Girls who consumed soda daily were 4.9 times more likely (Cl 2.8, 8.3; *p* < 0.001) to have reported being in a physical fight at least 12 times in the past month, whereas boys were 3.6 times more likely (CL 2.3, 5.8; *p* < 0.001) to have reported being in a physical fight at least 12 times in the past month. Girls who consumed soda every day were 20.9 times more likely (Cl 10.5, 41.7; *p* < 0.001) to consume fast food 7 times a week compared to males, who were 13.4 times more likely (Cl 7.3, 24.3; *p* < 0.001) to consume fast food 7 times a week. These same-sex differences in odds ratios were observed for daily ED consumption as well (Table 5).

## 4. Discussion

Our investigation explored the potential link between frequent soda and ED consumption and the propensity for engaging in risk behaviors in high-school students. In this analysis, we designated soda and ED consumption as our two predictor variables to examine their influence on these behaviors. The results were consistent with our hypothesis, revealing a positive relationship between daily consumption of both soda/EDs and behaviors such as vaping, alcohol use, weapon carrying, physical altercations, and reported suicide attempts. Both daily soda and ED consumption were linked to greater feelings of sadness or hopelessness and greater odds of suicide attempts, but neither beverage was directly related to self-reported mental health in the past month. Interestingly, there were mixed results between daily soda and ED consumption and relationships with sleep duration, with daily soda consumption associated with lower odds of insufficient sleep, but daily ED consumption associated with greater odds of insufficient sleep. Daily consumption of both drinks were linked to increased fast food consumption, suggesting a potential link to unhealthier dietary patterns. Sex differences emerged when examining specific behaviors, with girls who were daily soda and ED drinkers exhibiting a stronger association with negative outcomes like daily alcohol use and frequent physical fights. Although these findings are cross-sectional and cannot determine a causal relationship, they suggest that further investigation into the potential causal links between soda and ED consumption and these risky behaviors, particularly among adolescents, is warranted.

Daily consumption of soda and EDs was found to be strongly associated with substance use behaviors. These findings are similar to our earlier research, which focused solely on soda [20]. Previous studies have also shown links between ED use and substance use behaviors in adults [24,25] and adolescents [26,27]. Our study extends these findings by using a national sample and adding additional mental health and vaping outcomes that were not available in earlier studies. When taken together, these findings replicate and extend previous research, showing that soda and ED consumption is strongly positively associated with substance use, including alcohol, vaping, and other illicit drug use. The question remains whether there are causal links between soda and ED consumption and these behaviors, or whether they are coincidental. For example, it is possible that the caffeine contained within these drinks acts directly on the neural substrate that responds to substance use, thereby increasing a desire to use illicit drugs. It is also possible that these behaviors are linked indirectly, with teens who are generally higher in sensation seeking being more likely to engage in risk behaviors and to consume soda and EDs regularly.

We also found relationships between the consumption of caffeine and violent acts, such as physical fights and carrying a weapon to school. This replicates the findings from our previous study that examined only soda consumption [20]. In the current study, we found that daily soda and ED use was associated with a greater likelihood of both carrying a weapon to school and getting into a physical fight. Research conducted by Scalese et al., also concluded that the consumption of EDs and alcohol-mixed EDs were both associated with physical violence in teenagers [26]. Finally, a longitudinal study by Kristjansson et al., (2021) showed that caffeine consumption at baseline was positively associated with aggressive behavior one year later in adolescents [28]. This shows that there may be a causal relationship between caffeine consumption and aggressive behavior, but more work needs to be carried out to replicate and extend these analyses to other types of risk behaviors and consider variations in beverage types and potential moderating factors like sex. By employing longitudinal study designs, similar to Kristjansson et al.’s approach, researchers can understand the temporal and causal pathways that underlie the relationship between caffeinated beverage consumption and risky behaviors.

We also examined relationships between soda and ED consumption with mental health outcomes. Unlike the other analyses, our findings here were mixed. The daily intake of soda and EDs raised the likelihood of experiencing depression or hopelessness as well as the number of suicide attempts that were reported; however, there was no relationship between reporting “Mental health not good in past 30 days” and soda or ED consumption. Previous studies have shown that greater caffeine intake is associated with internalizing behavior symptoms such as anxiety, depression, and psychosomatization [28,29]. A study similar to ours conducted in New Zealand adolescents found that greater energy drink consumption was associated with greater depressive symptoms, mental health difficulties, and poorer overall well-being [30]. A review by Richards and Smith (2016) of studies in both adults and adolescents suggests that the literature is mixed, but in general, chronic caffeine consumption is associated with poorer mental health outcomes [10]. While the current study identified relationships between daily soda and ED consumption and sadness/ hopelessness and suicide attempts, it did not find a clear association with overall self-reported mental health. This may represent a trend in adolescents to acknowledge poorer general mental health than in the past. Indeed, in this sample, the majority of respondents, regardless of consumption frequency, reported at least some degree of poor mental health. These findings, while somewhat contradictory to existing research, highlight the need for more current research that represents changing trends in both beverage consumption and other health behaviors. Our ability to examine these relationships is also limited by the relatively weak assessment of mental health outcomes in the YRBS. A more robust analysis of mental health needs to be included in the updated YRBS and should be considered in all states and not just a subset.

Daily consumption of soda and EDs was linked to some, but not all, of the lifestyle behaviors that we examined. Daily ED consumption was associated with shorter sleep duration, although there was no clear relationship between the consumption of soda and reduced sleep duration. Shorter sleep duration and quality have been linked to greater caffeinated beverage consumption in a number of previous studies in adolescents and young adults [31,32,33]. In fact, insomnia is one of the most consistent side-effects of caffeinated beverage consumption. In the current study, less than 8% of adolescents, in any consumption category, reported getting the recommended amount of sleep per night. In addition, when we examined sleep duration as a continuous variable in the ANOVA analysis, we did find a significant difference among the soda consumption groups, with shorter average weeknight sleep in the daily group compared with the occasional and soda non-consumers. It should be noted that the averages were far below the recommended 9 h of sleep, with all three groups being at 6.5 h or below. Further research is needed to explore this inconsistency and to elucidate the specific factors influencing these relationships. In addition, studies that employ objective measures of sleep instead of relying on self-report can provide more accurate data related to these relationships. In addition to sleep, we also found that daily soda and ED consumption were significantly associated with greater intake of fast food and that the odds were two to three times higher for soda and EDs. This is similar to findings from a study by Almulla and Faris (2020), who found that ED consumption was associated with both reduced sleep and increased energy-dense food consumption in adolescents from the UAE [34]. One possible explanation for this is that fast food establishments are a common source of soda for consumers. It may also be part of a pattern of less healthy food and beverage consumption.

Finally, we examined how the relationships described above were influenced by sex. In general, we discovered that boys were more likely than girls to be daily consumers of both soda and EDs. This is consistent with previous studies showing sex differences in caffeine consumption patterns [9,35,36]. However, when girls did regularly consume soda and EDs, the likelihood of them participating in risky behaviors, such as drinking alcohol, getting into physical altercations, and consuming fast food, was higher than that of boys at the same level of consumption. When considered collectively, these results imply that there might be sex-dependent variations in the motivations and consequences of beverage intake. Specifically, although girls rarely consume soda and ED daily, when they do, it appears to be strongly associated with other risky behaviors. Again, a causal link cannot be established here, but this relationship may be relevant for future interventions aimed at educating youth about caffeine consumption and mitigating the risks associated with caffeine consumption in adolescents.

### Strengths and Limitations

This study benefits from several strengths that enhance the generalizability and informativeness of its findings. First, it leverages a large sample that encompasses participants from various states, increasing the study’s representativeness of the national population. Second, it incorporates recent data on ED consumption and risk-taking behaviors, such as vaping, which represents a novel contribution to the existing research in this field. Furthermore, the study design ensured a balanced participation rate between boys and girls, along with representation from diverse racial/ethnic groups and socioeconomic backgrounds (SES). This study was not without limitations. First, these data are cross-sectional and correlational; thus, causal links between ED and soda consumption and risk behaviors cannot be established from these findings. More longitudinal, prospective studies need to be conducted to interpret casual relationships. Second, since the data rely on self-reporting, it is susceptible to potential biases and inaccuracies. Third, the surveys did not distinguish between caffeinated soda and non-caffeinated soda consumption. Thus, while all EDs are highly caffeinated and the consumption of these can be a proxy for caffeine consumption, the soda intake is not a direct index of caffeine intake in this sample. Fourth, the lack of access to physiological or medical records further restricts the ability to verify self-reported information on drug use, caffeine and alcohol intake, sleep behaviors, and mental health diagnoses. Fifth, the YRBS did not assess other potential confounders, such as socioeconomic status, parental influence, or school environment. Finally, the YRBS did not collect details on the timing of caffeine consumption, which could have provided a more nuanced understanding of its correlation with sleep patterns, among other behaviors.

## 5. Conclusions

This study showed that daily soda and ED consumption in adolescents was associated with greater risk-taking behaviors such as vaping, alcohol and substance use, weapon carrying, and physical altercations. Daily consumption was associated with increased odds of reported suicide attempts, particularly for ED consumption. These findings replicate and extend our previous work [19] and that of others, and highlight a worrying association between soda and ED consumption frequency and adolescent risk-taking behaviors, mental health, and other health behaviors. Future research should investigate the potential causality of these associations and how these beverages impact other aspects of adolescent health and well-being, given their growing popularity. From there, harm reduction approaches can be used to educate adolescents and parents and, hopefully, limit adolescent caffeinated beverage consumption. To our knowledge, there are no public policies in the US aimed at reducing soda or ED consumption in youth. If causal links are established between soda and ED consumption and risk behaviors, this evidence could put pressure on law makers to institute common-sense policies, such as age limits on energy drink purchasing and public health messaging about the potential harms of the consumption of highly caffeinated beverages.

## Figures and Tables

**Table 1:** Study participant characteristics.

	All		No Soda		Occasional Soda		Daily Soda		*p*	No ED		Occasional ED		Daily ED		*p*
	N	%	N	%	N	%	N	%		N	%	N	%	N	%	
Sex									<0.001							<0.001
Female	5442	51.6	2053	37.7	2818	51.8	571	10.5		3698	69.8	1320	24.9	277	5.2	
Male	5106	48.4	1381	27.0	2811	55.1	914	17.9		2779	55.8	1691	33.9	507	10.2	
Age									<0.001							<0.001
<14	1248	11.8	394	11.4	678	12.0	176	11.8		739	61.1	384	31.8	86	7.1	
15	2826	26.7	883	25.6	1555	27.5	388	25.9		1739	62.9	809	29.3	215	7.8	
16	2827	26.7	847	24.5	1515	26.8	365	24.4		1747	63.8	782	28.6	209	7.6	
17	2378	22.4	806	23.4	1234	21.8	338	22.6		1487	64.2	658	28.4	171	7.4	
>18	1324	12.4	421	12.2	675	11.9	228	15.3		797	61.2	391	30.1	113	8.7	
Grade									<0.001							<0.001
9th	2937	27.9	909	26.6	1612	28.7	416	28.1		1721	60.3	914	32.0	219	7.7	
10th	2862	27.2	900	26.3	1557	27.7	405	27.4		1797	64.1	775	27.7	230	8.2	
11th	2633	25.1	900	26.3	1380	24.5	353	23.9		1621	63.9	736	29.0	178	7.0	
12th	2087	19.8	708	20.7	1073	19.1	306	20.7		1318	64.1	584	28.4	155	7.5	
Race/Ethnicity									<0.001							<0.001
American Indian/Alaskan Native	253	2.4	52	1.5	159	2.8	42	2.9		144	57.4	79	31.5	28	11.2	
Asian	314	3.0	138	4.1	149	2.7	27	1.8		243	78.9	54	17.5	11	3.6	
Black/African American	1098	10.5	377	11.1	548	9.8	173	11.8		517	52.3	339	34.3	133	13.4	
Hawaiian/Pacific Islander	55	0.5	13	0.4	32	0.6	10	0.7		24	46.2	22	42.3	6	11.5	
White/Caucasian	5241	50.2	1682	49.5	2825	50.6	734	49.9		3537	67.6	1407	26.9	285	5.5	
Hispanic/Latino	428	4.1	134	3.9	239	4.3	55	3.7		221	55.8	141	35.6	34	8.6	
Multiple Race–Hispanic	2543	24.3	821	24.2	1357	24.3	365	24.8		1398	57.1	802	32.8	248	10.1	
Multiple Race–Non-Hispanic	515	5.0	178	5.2	271	4.9	66	4.5		331	65.4	138	27.3	37	7.3	
	**Mean**	**SEM**	**Mean**	**SEM**	**Mean**	**SEM**	**Mean**	**SEM**	***p***	**Mean**	**SEM**	**Mean**	**SEM**	**Mean**	**SEM**	
BMI Percentile	61.6	0.29	61.6	0.49	61.9	0.39	60.1	0.29	0.12	60.8	0.37	62.9	0.54	62.1	1.1	0.005
Estimated Caffeine Use (mg/Day)	64.5	1.15	21.7 ^a^	1.20	51.9 ^b^	0.96	209.2 ^c^	5.2	<0.001	16.8	0.34	84.5	0.78	381.7	7.5	<0.001
Hours of Sleep/Night	6.45	0.01	6.43 ^a^	0.03	6.51 ^b^	0.02	6.29 ^c^	0.04	<0.001	6.5	0.02	6.4	0.03	6.3	0.06	<0.001

Participant characteristics as a function of soda and energy drink consumption. *p* values represent differences in each category as assessed by Chi-squared or ANOVA. Different letters (^a,b,c^) indicate significant difference from one another (*p* < 0.001).

**Table 2:** Substance use behavior as a function of soda and ED consumption.

	Soda					Energy Drinks				
	Daily Soda	No Soda	Daily vs. No Soda			Daily EDs	No EDs	Daily vs. No EDs		
	N	N	Odds Ratio	(95% CI)	*p*	N	N	Odds Ratio	(95% CI)	*p*
Cigarette Smoking in past 30 days										
Everyday	52	21	5.95	(3.6, 9.9)	<0.001	40	42	8.36	(5.3, 13.1)	<0.001
20–29 Days	9	3	10.02	(2.2, 47.3)	0.004	6	6	7.65	(2.3, 25.2)	<0.001
10–19 Days	14	11	2.95	(1.3, 6.6)	0.009	10	23	4.23	(2.0, 8.9)	<0.001
1–9 Days	73	100	1.82	(1.3, 2.5)	<0.001	38	180	1.89	(1.3, 2.7)	<0.001
0 Days	1300	3263	ref	ref	ref	667	6173	Ref	ref	ref
Vape Use in past 30 days										
Everyday	80	39	7.27	(4.8, 11.1)	<0.001	38	134	10.92	(6.8, 17.5)	<0.001
20–29 Days	25	32	2.99	(1.7, 5.2)	<0.001	4	77	1.98	(0.7, 5.6)	0.20
10–19 Days	24	32	2.54	(1.4, 4.4)	0.001	12	90	4.65	(2.3, 9.3)	<0.001
1–9 Days	51	92	1.94	(1.3, 2.8)	<0.001	18	240	2.83	(1.6, 4.9)	<0.001
0 Days	203	704	ref	ref	ref	50	1757	Ref	ref	ref
Alcohol Use in past 30 days										
Everyday	47	20	6.41	(3.7, 11.0)	<0.001	45	20	19.54	(11.4, 33.6)	<0.001
20–29 Days	16	12	3.59	(1.7, 7.6)	<0.001	7	18	3.62	(1.5, 8.7)	0.004
10–19 Days	44	33	3.52	(2.2, 5.6)	<0.001	26	67	3.67	(2.3, 5.8)	<0.001
1–9 Days	489	1013	1.29	(1.1, 1.5)	<0.001	209	1975	0.99	(0.8, 1.2)	0.90
0 Days	823	2231	ref	ref	ref	452	4196	Ref	ref	ref
Marijuana Use in past 30 days										
40+ Times	123	57	5.54	(3.9, 7.7)	<0.001	83	148	5.11	(3.8, 6.8)	<0.001
20–39 Times	28	31	2.32	(1.4, 3.9)	0.002	10	73	1.26	(0.6, 2.5)	0.495
10–19 Times	46	56	2.09	(1.4, 3.1)	<0.001	31	106	2.56	(1.7, 3.9)	<0.001
1–9 Times	270	762	0.87	(0.7, 1.0)	0.079	129	1455	0.80	(0.6, 0.9)	0.034
0 Times	996	2500	ref	ref	ref	520	4673	Ref	ref	ref
Prescription Drugs in lifetime										
40+ Times	59	35	3.71	(2.4, 5.7)	<0.001	48	54	6.87	(4.6, 10.3)	<0.001
20–29 Times	21	15	3.44	(1.7, 6.8)	<0.001	18	24	6.05	(3.3, 11.2)	<0.001
10–19 Times	30	42	1.71	(1.1, 2.8)	0.031	18	66	2.11	(1.2, 3.6)	0.007
1–9 Times	152	239	1.47	(1.2, 1.8)	<0.001	83	480	1.35	(1.1, 1.7)	0.021
0 Times	1101	2541	ref	ref	ref	586	4749	Ref	ref	ref

**Table 3:** Relationship among soda and ED consumption and mental health outcomes.

	Soda				EDs			
	Daily	None	Odds Ratio (95% CI)	*p*	Daily	None	Odds Ratio (95% CI)	*p*
Sad or hopeless almost every day for 2 weeks (past 12 months)				<0.001				<0.001
Yes	606	876	1.3 (1.1, 1.5)		297	2234	1.4 (1.2, 1.6)	
No	1174	2230	ref		487	4203	ref	
Suicide attempt (past 12 months)								
6+ Times	30	15	5.5 (2.8, 10.7)	<0.001	33	24	13.0 (7.3, 23.3)	<0.001
4–5 Times	6	14	1.3 (0.5, 3.5)	0.58	3	21	5.7 (2.9, 11.2)	<0.001
2–3 Times	46	64	1.8 (1.2, 2.7)	0.003	32	126	4.4 (2.2, 8.9)	<0.001
1 Time	95	128	1.9 (1.5, 2.6)	<0.001	43	225	8.1 (2.0, 31.6)	<0.001
0 Times	1098	2852	ref	ref	521	5555	ref	ref
Mental health not good (past 30 days)								
All 30 Days	11	35	1.1 (0.51, 2.3)	0.81	7	81	0.54 (0.19, 1.5)	0.23
14–29 Days	15	79	0.65 (0.34, 1.2)	0.19	2	134	2.8 (0.64, 12.6)	0.17
7–13 Days	10	88	0.35 (0.16, 0.75)	0.007	2	151	2.7 (0.77, 9.3)	0.12
1–6 Days	47	203	0.75 (0.47, 1.2)	0.22	9	411	1.9 (0.72, 4.8)	0.20
0 Days	47	151	ref	ref	19	343	ref	ref

**Table 4:** Relationship among soda and ED consumption and lifestyle behaviors.

	Soda				EDs			
	Daily	None	Odds Ratio (95% CI)	*p*	Daily	None	Odds Ratio (95% CI)	*p*
Sleep								
<5 h	447	917	0.88 (0.68, 1.14)	0.34	145	348	0.8 (0.6, 1.1)	0.25
6 h	370	754	0.87 (0.67, 1.13)	0.30	273	1193	1.7 (1.2, 2.2)	<0.001
7 h	345	926	0.66 (0.51, 0.86)	0.002	174	1863	2.1 (1.6, 2.9)	<0.001
8 h	201	593	0.61 (0.46, 0.81)	<0.001	111	2423	2.1 (1.5, 2.9)	<0.001
>9 h	113	203	ref	ref	76	348	ref	ref
Fruit (per week)								
0 times	247	534	1.07 (0.88, 1.3)	0.48	94	898	1.9 (1.4, 2.5)	<0.001
1–3 times	459	1003	1.06 (0.89, 1.3)	0.49	173	2275	2.3 (1.8, 2.9)	<0.001
4–6 times	238	699	0.78 (0.65, 0.95)	0.014	127	1411	1.9 (1.5, 2.6)	<0.001
7 times	188	368	1.18 (0.95, 1.46)	0.13	104	687	1.2 (0.92, 1.6)	0.18
8 or more times	366	843	ref	ref	122	1230	ref	ref
Vegetables (per week)								
0 times	402	718	1.12 (0.97, 1.45)	0.09	179	1345	2.0 (1.6, 2.5)	<0.001
1–3 times	431	1073	0.85 (0.69, 1.03)	0.09	203	2264	2.9 (2.3, 3.6)	<0.001
4–6 times	254	741	0.73 (0.59, 0.90)	0.004	125	1440	2.9 (2.3, 3.7)	<0.001
7 times	173	428	0.84 (0.66, 1.06)	0.14	105	727	1.8 (1.4, 2.4)	<0.001
8 or more times	231	485	ref	ref	184	733	ref	ref
Fast Food (per week)								
7 times	89	33	17.5 (11.2, 27.4)	<0.001	66	50	10.7 (6.9, 16.6)	<0.001
5–6 times	54	34	10.7 (6.6, 17.3)	<0.001	41	70	6.1 (4.1, 9.3)	<0.001
2–4 times	259	224	7.6 (5.8, 9.9)	<0.001	160	472	3.4 (2.2, 5.2)	<0.001
1–2 times	344	678	3.3 (2.6, 4.3)	<0.001	228	1184	1.84 (1.1, 3.2)	<0.001
0 times	114	731	ref	ref	101	884	ref	ref
Physical Activity (2 h per day)								
0 days	429	909	1.03 (0.84, 1.28)	0.76	349	1293	1.8 (1.2, 2.8)	0.009
1–2 days	634	1318	1.07 (0.87, 1.31)	0.54	304	2414	1.3 (0.85, 1.98)	0.23
3–4 days	118	377	0.69 (0.52, 0.90)	0.007	39	884	0.42 (0.31, 0.94)	<0.001
5–6 days	133	422	0.69 (0.53, 0.91)	0.008	33	993	0.22 (0.16, 0.29)	<0.001
7 days	175	393	ref	ref	61	892	ref	ref

**Table 5:** Sex effects on relationships among soda and ED consumption and various health behaviors.

	Boys				Girls			
	Daily Soda	No Soda	Daily Adjusted Odds Ratio		Daily Soda	No Soda	Daily Adjusted Odds Ratio	
	N	N	(95% CI)	*p*	N	N	(95% CI)	*p*
Vape Use (past 30 days)								
Everyday	60	17	6.9 (3.9, 12.4)	<0.001	18	22	6.6 (3.3, 13.1)	<0.001
20–29 Days	18	14	2.4 (1.2, 5.1)	0.017	6	17	3.1 (1.1, 8.2)	0.027
10–19 Days	17	13	2.6 (1.2, 5.5)	0.014	6	19	2.4 (0.9, 6.1)	0.081
1–9 Days	31	29	2.0 (1.2, 3.5)	0.011	20	63	2.2 (1.2, 4.0)	0.008
0 Days	142	268	ref	ref	60	428	ref	ref
Alcohol Use (past 30 days)								
Everyday	29	16	3.3 (1.8, 6.2)	<0.001	14	4	17.8 (5.0, 62.9)	<0.001
20–29 Days	10	6	3.1 (1.1, 8.7)	0.28	6	6	4.0 (1.3, 12.6)	0.017
10–19 Days	32	15	3.8 (2.0, 7.2)	<0.001	10	18	2.2 (1.0, 4.9)	0.044
1–9 Days	280	345	1.5 (1.2, 1.8)	<0.001	206	661	1.3 (1.0, 1.5)	0.024
0 Days	500	921	ref	ref	317	1295	ref	ref
Marijuana Use (past 30 days)								
40+ Times	88	31	5.1 (3.3, 7.9)	<0.001	32	24	4.8 (2.8, 8.3)	<0.001
20–39 Times	17	14	2.3 (1.1, 4.7)	0.029	11	17	2.3 (1.1, 5.2)	0.037
10–19 Times	30	21	2.3 (1.3, 4.1)	0.004	16	34	1.9 (1.0, 3.5)	0.042
1–9 Times	151	264	0.96 (0.78, 1.2)	0.715	117	497	0.87 (0.69, 1.1)	0.246
0 Times	599	1022	ref	ref	387	1457	ref	ref
Been in a Physical Fight (past 30 days)								
12+ Times	56	30	3.6 (2.3, 5.8)	<0.001	14	12	4.9 (2.3, 11.0)	<0.001
8–11 Times	11	9	2.3 (0.93, 5.5)	0.072	8	8	3.9 (1.4, 10.7)	0.010
4–7 Times	39	37	2.0 (1.2, 3.3)	0.004	18	24	3.6 (1.9, 6.7)	<0.001
1–3 Times	216	212	1.9 (1.5, 2.3)	<0.001	107	163	3.1 (2.4, 4.1)	<0.001
0 Times	573	1060	ref	ref	416	1826	ref	ref
Sleep (per school night)								
<5 h	241	349	0.92 (0.64, 1.3)	0.624	201	557	1.1 (0.73, 1.7)	0.62
6 h	222	272	1.1 (0.73, 1.5)	0.792	145	479	0.93 (0.61, 1.4)	0.76
7 h	222	372	0.78 (0.6, 1.1)	0.172	118	549	0.66 (0.42, 1.0)	0.058
8 h	137	260	0.69 (0.48, 1.0)	0.052	64	328	0.60 (0.38, 0.97)	0.036
>9 h	75	98	ref	ref	34	105	ref	ref
Fast Food (per week)								
7 times	48	19	13.4 (7.3, 24.3)	<0.001	36	14	20.9 (10.5, 41.7)	<0.001
5– 6 times	23	14	8.8 (4.3, 18.1)	<0.001	31	20	13.3 (6.9, 25.3)	<0.001
3–4 times	141	85	8.5 (5.8, 12.5)	<0.001	116	135	7.0 (4.7, 10.4)	<0.001
1–2 times	211	257	4.3 (3.1, 5.9)	<0.001	129	419	2.6 (1.8, 3.7)	<0.001
0 times	65	326	ref	ref	48	398	ref	ref
	**Boys**				**Girls**			
	**Daily ED**	**No ED**	**Daily Adjusted Odds Ratio**		**Daily ED**	**No ED**	**Daily Adjusted Odds Ratio**	
	**N**	**N**	**(95% CI)**	***p***	**N**	**N**	**(95% CI)**	***p***
Vape Use (past 30 days)								
Everyday	28	69	10.3 (5.9, 18.1)	<0.001	8	65	7.2 (2.9, 18.0)	<0.001
20–29 Days	2	34	2.9 (1.5, 6.1)	0.003	2	41	3.8 (1.1, 13.4)	0.036
10–19 Days	10	37	1.5 (0.66, 3.5)	0.33	1	52	5.9 (0.7, 48.7)	0.10
1–9 Days	14	99	6.6 (1.5, 29.6)	0.013	4	140	2.5 (0.5, 12.6)	0.26
0 Days	35	837	ref	ref	15	903	ref	ref
Alcohol Use (past 30 days)								
Everyday	26	17	9.6 (5.1, 17.9)	<0.001	15	3	69.1 (19.8, 241.0)	<0.001
20–29 Days	4	7	8.3 (4.3, 15.7)	<0.001	3	11	81.5 (23.1, 287.9)	<0.001
10–19 Days	22	34	2.2 (0.98, 5.0)	0.057	2	33	82.2 (12.4, 544.1)	<0.001
1–9 Days	134	762	2.6 (0.7, 10.2)	0.18	73	1204	18.3 (3.1, 108.5)	0.001
0 Days	283	1834	ref	ref	167	2325	ref	ref
Marijuana Use (past 30 days)								
40+ Times	61	81	4.6 (3.2, 6.5)	<0.001	19	65	4.2 (2.5, 7.1)	<0.001
20–39 Times	8	28	5.8 (3.8, 8.7)	<0.001	2	45	4.9 (2.7, 8.8)	<0.001
10–19 Times	21	49	1.8 (0.99, 3.4)	0.053	10	57	1.7 (0.73, 3.9)	0.22
1–9 Times	74	581	2.6 (1.1, 6.2)	0.027	53	872	6.7 (1.5, 30.1)	0.014
0 Times	328	1997	ref	ref	186	2632	ref	ref
Been in a Physical Fight (past 30 days)								
12+ Times	40	56	5.7 (3.8, 8.8)	<0.001	16	16	17.3 (8.5, 35.2)	<0.001
8–11 Times	8	18	2.3 (1.5, 3.7)	<0.001	4	13	6.1 (2.9, 13.0)	0.010
4–7 Times	28	56	1.5 (0.8, 2.7)	0.21	9	49	5.4 (2.0, 14.6)	<0.001
1–3 Times	131	423	1.9 (0.7, 4.9)	0.20	53	327	3.2 (0.87, 12.1)	0.081
0 Times	277	2190	ref	ref	187	3269	ref	ref
Sleep (per school night)								
<5 h	137	588	0.89 (0.60, 1.3)	0.59	106	925	1.1 (0.67, 1.8)	0.72
6 h	114	583	1.6 (1.1, 2.3)	0.01	55	906	2.0 (1.2, 3.5)	0.008
7 h	116	842	2.1 (1.4, 3.0)	<0.001	54	1008	2.3 (1.4, 3.9)	0.002
8 h	77	547	1.99 (1.3, 3.0)	<0.001	34	640	2.3 (1.3, 4.1)	0.004
>9 h	50	175	ref	ref	22	171	ref	ref
Fast Food (per week)								
7 times	42	20	11.7 (6.5, 21.3)	<0.001	19	29	9.4 (4.8, 18.4)	<0.001
5–6 times	19	25	6.3 (3.6, 11.0)	<0.001	22	44	6.1 (3.3, 11.4)	<0.001
3–4 times	96	161	3.5 (2.0, 6.5)	<0.001	62	309	3.2 (1.7, 6.0)	<0.001
1–2 times	148	446	2.8 (1.2, 6.2)	0.012	78	732	1.3 (0.6, 2.8)	0.51
0 times	66	364	ref	ref	35	510	ref	ref

## Data Availability

The YRBS data are publicly available (https://www.cdc.gov/yrbs/data/index.html).
